# Synthesis of Steryl Hydroxycinnamates to Enhance Antioxidant Activity of Rapeseed Oil and Emulsions

**DOI:** 10.3390/ma13204536

**Published:** 2020-10-13

**Authors:** Dobrochna Rabiej-Kozioł, Marek P. Krzemiński, Aleksandra Szydłowska-Czerniak

**Affiliations:** Faculty of Chemistry, Nicolaus Copernicus University in Toruń, Gagarina 7, 87-100 Toruń, Poland; d.rabiej@umk.pl (D.R.-K.); mkrzem@umk.pl (M.P.K.)

**Keywords:** hydroxycinnamate steryl esters, antioxidant activity, oils, emulsions, synthesis

## Abstract

In recent years, steryl esters have found potential applications in food, pharmaceutical and cosmetic industries. Therefore, three hydroxycinnamate steryl esters (HSEs): β-sitosteryl sinapate (β-SSA), β-sitosteryl caffeate (β-SCA), and β-sitosteryl ferulate (β-SFA) were synthesized by chemical approach and their antioxidant activity (AA) were analyzed by 2,2-diphenyl-1-picrylhydrazyl (DPPH) and 2,2′-azinobis-(3-ethylbenzothiazoline-6-sulphonic acid) (ABTS) assays. The values of inhibitory concentration (IC_50_) of each ester needed to inhibit 50% of the DPPH radical (IC_50(DPPH)_ = 238.9, 78.3, 290.0 µmol/L for β-SSA, β-SCA, and β-SFA, respectively) and ABTS radical cation (IC_50(ABTS)_ = 174.6, 106.7, 206.0 µmol/L for β-SSA, β-SCA, and β-SFA, respectively) were estimated and compared with antioxidant potential of phenolic acids. Moreover, the effect of HSEs addition in the concentrations range between 0.01% and 0.5% on the AA of refined rapeseed oil, mayonnaise and margarine was evaluated. Chemical structures of the synthesized HSEs and their concentrations strongly affect the AA of fat products. Oil and emulsions supplemented with higher concentrations of HSEs had significantly higher AA than control samples. Unfortunately, lower concentrations of HSEs (0.01% and 0.02%) did not increase the AA of fat products. However, steryl phenolates added in higher amounts can be considered as potential antioxidants delaying the oxidation processes of studied fats.

## 1. Introduction

Phenolic acids naturally present in plants play important role in food supplementation due to their antioxidant potential. Antioxidant properties of phenolic acids derivatives can be predicted by specific structure-activity relationships (SAR). Lipophilic, antioxidant and biological functions of phenolic acids depend on the number of hydroxyl groups in the aromatic ring and orto-substitution with the electron donor methoxy group, which increases the stability of the phenoxy radical [1,2,3,4]. Phenolic acids can be divided into two major groups such as hydroxycinnamic acids (HCA) and the hydroxybenzoic acids (HBA). The main HCA: p-coumaric (p-CouA), caffeic (CA), ferulic (FA), and sinapic (SA) acids and their derivatives are natural and the most common bioactive compounds in plants, oilseeds and cereals [4,5]. Among them hydroxycinnamate steryl esters (HSEs), mainly esterified to esters of p-CouA or FA are present in plants and plant-derived foods. It is known that plant phytosteryl esters possess biological activities including antioxidant potential, anti-inflammatory activity, and antitumor activity as well as anti-diabetic, anti-ageing, neuroprotective, hepatoprotective, and cholesterol-lowering properties [1].

Steryl ferulates (FA esters of sterols) are bioactive compounds identified in an inner pericarp and aleurone layer of cereals grains such as: rice, wheat, rye, corn, and triticale. The mixture of steryl ferulates: 4,4′-dimethyl sterol esters 24-methylenecycloartanyl ferulate and cycloartenyl ferulate naturally present in rice is known as γ-oryzanol, while desmethylsterols (sitosterol, campesterol and their saturated forms, stanols) are dominated in other cereals [6,7,8,9,10]. Moreover, steryl caffeates (campesterol and cycloartenol caffeates) as well as steryl sinapate (24-methylenecholesteryl sinapate) were found in various rice varieties [11,12,13].

On the other hand, HSEs can be important antioxidant additives in the fat industry due to their higher solubility in oils caused by hydrophobic moiety of sterols and lower melting points compared with free natural phytosterols [14].

Additionally, higher molecular weight of HSEs in comparison with phenolic acids limits their evaporation and thermal decomposition at high temperatures during frying, boiling, and technological processes [15,16]. Therefore, HSEs are more effective inhibitors of oxidation processes at high temperatures in a long time and can be considered better antioxidants than free phenolic acids for fat industry. Corn steryl ferulates demonstrated protective effect on endogenous tocopherols in soybean oil during frying, while γ-oryzanol was faster degraded at high temperature in this process [16]. On the other hand, the highest oxidative stability had soybean oil after the FA addition, whereas lower oxidation stability indexes were determined for an oil with sitostanol ferulate and sitostanol [17]. However, lipophilic antioxidants, such as phenolic acids esters, are more active in emulsions, whereas hydrophilic antioxidants such as phenolic acids are more efficient in oils according to “polar paradox” theory. Therefore, esterification of sterols with phenolic acids is an effective method for the application of these hydrophilic antioxidants in lipophilic systems in order to improve antioxidant properties and oxidative stability [18,19].

It is known that structural variations of phytosteryl esters affect their antioxidant activity (AA) [16]. Currently, different analytical methods: 2,2′-azinobis-(3-ethylbenzothiazoline-6-sulphonic acid) (ABTS), 2,2-diphenyl-1-picrylhydrazyl (DPPH), cupric reducing antioxidant capacity (CUPRAC), oxygen radical absorbance capacity (ORAC), β-carotene bleaching, and reducing power assay have been used for analysis of antioxidant properties of phytosteryl esters [4,6,16,20,21,22,23]. However, degradation products and oxidative stability of edible oils enriched with phytosteryl esters were analyzed using chromatographic methods and Rancimat test, respectively [15,16,23].

In general, chemical, enzymatic or chemoenzymatic esterifications, and transesterification have been often proposed for the synthesis of phytosteryl esters of fatty acids, whereas the esterification of phytosterols with phenolic acids were reported only by some authors [4,6,7,8,16,20,21,22,23]. Recently, microwave and ultrasound irradiations have been successfully applied in esterification of phytosteryl esters [6,24]. Steryl ferulates were synthesized by different pathways, while an enzymatic and chemoenzymatic methods for esterification of phytosterols with CA, CouA, SA, and vanillic acid (VA) were mainly used [4,6,7,8,16,20,21,22,23].

Although various by-products (dehydrated compounds, oxysterols) can be generated during chemical synthesis of phytosteryl esters, this method is preferred for the commercial production of phytosteryl esters due to the high cost of enzyme and lower productivity of enzymatic synthesis.

For this reason, the present work was focused on a chemical method to synthesis of three HSEs: β-sitosteryl sinapate (β-SSA), β-sitosteryl caffeate (β-SCA), and β-sitosteryl ferulate (β-SFA). The chemical structures of the synthesized and purified HSEs were determined and confirmed by ^1^H and ^13^C NMR spectroscopy. Furthermore, the modified DPPH and ABTS assays were applied to determine the antioxidant potential of synthesized steryl hydroxycinnamates and to compare their AA with antioxidant efficacy of phenolic acids. Finally, the effect of concentration of the obtained HSEs on radical scavenging activity of the refined rapeseed oil and water-in-oil (w/o) and oil-in-water (o/w) emulsions: margarine and mayonnaise, respectively was also evaluated.

## 2. Material and Methods

### 2.1. Reagents

Phenolic acids: SA, CA, and FA, β-sitosterol ≥ 70% (mainly impurities campesterol and β-sitostanol), 2,2-diphenyl-1-picrylhydrazyl radical (DPPH, 95%), 2,2′-azino-bis(3-ethylbenzothiazoline-6-sulfonic acid) diammonium salt (ABTS), acetic anhydride, 4-(dimethylamino)pyridine (DMAP), 6-hydroxy-2,5,7,8-tetramethylchromane-2-carboxylic acid (Trolox), dicyclohexylcarbodiimide (DCC), potassium persulfate and potassium carbonate were obtained from Merck, Warszawa, Poland. Methanol, ethanol, ethyl acetate, dichloromethane, chloroform, hexane, and hydrochloric acid were purchased from Chempur, Piekary Śląskie, Poland. Thin layer chromatography (TLC) plates with fluorescent indicator UV_254_, trade name ALUGRAM R SIL G/UV_254_ (Macherey-Nagel, Dueren, Germany) and silica gel (pore size 60Å, 230–400 mesh, Kieselgel, Macherey-Nagel, Dueren, Germany) were purchased from Alchem, Toruń, Poland.

### 2.2. Samples

The refined rapeseed oil in the original packaging (polyethylene terephthalate (PET) bottle) was kindly provided by a local vegetable oil factory. Margarine and mayonnaise originally packed into PET box and glass jar, respectively were purchased from local. All fat samples were within their stated shelf lives and stored in a refrigerator until analysis.

### 2.3. NMR Analysis

The nuclear magnetic resonance (NMR) spectroscopy was used for confirmation of the chemical structures of the synthesized HSEs. ^1^H and ^13^C NMR of pure compounds from each step of synthesis were measured at 298 ± 1 K on a Bruker Avance III 700 MHz and 400 MHz spectrometers (Bruker Corporation, Karlsruhe, Germany) operating at 700 or 400 MHz and 170 or 100 MHz, respectively. The samples were dissolved in CDCl_3_ and spectra were referenced to the solvent residual peak. Chemical shifts (δ) were reported as parts per million (ppm) and coupling constants (*J*) were given in hertz (Hz).

### 2.4. Chemical Synthesis

Esters of β-sitosterol with HCA were synthesized according to the procedure described by Winkler-Moser et al. [16].

#### 2.4.1. Acetylation of SA, CA, and FA to Its 4-OH or 3,4-OH Protected Derivatives

In a 100 mL three neck round bottom flask equipped with a dropping funnel, thermometer, magnetic stirrer, and reflux condenser connected to nitrogen line, SA (2.242 g, 10 mmol) was dissolved in pyridine (10 mL). DMAP (13 mg, 0.1 mmol) was added and the solution was cooled in an ice-bath to 0 °C under nitrogen. Then, acetic anhydride (1.123 g, 11 mmol) was added dropwise and stirring was continued for 15 min at 0 °C and for further 4 h at room temperature. Cold water (10 mL) was added to the flask and pyridine was neutralized by slow addition of 2 M HCl at 0 °C. The formed precipitate was filtered off on Büchner funnel and washed with water. Methanol (20 mL) was added to the crude product and the mixture was heated to reflux for 10 min and then stirred for 1 h at room temperature. The product was filtered and dried to give 4-O-acetylsinapic acid (2.397 g, yield 90%). ^1^H NMR (700 MHz, CDCl_3_) δ ppm: 2.35 (s, 3H), 3.86 (s, 6H), 6.40 (d, *J* = 15.9 Hz, 1H), 6.80 (s, 2H), 7.72 (d, *J* = 15.9 Hz, 1H). ^13^C NMR (100 MHz, CDCl_3_) δ ppm: 20.39, 56.19 (2 × OCH_3_), 104.94 (2 × CH), 117.54, 130.79, 132.28, 146.72, 152.47 (2 × C-OMe), 168.44, 172.02.

3,4-O-Diacetylcaffeic acid was synthesized applying the above procedure starting from CA (2.702 g, 15 mmol), DMAP (37 mg, 0.3 mmol), and acetic anhydride (3.369 g, 33 mmol). Purification procedure gave 3,4-O-diacetylcaffeic acid (3.250 g, yield 82%). ^1^H NMR (700 MHz, CDCl_3_) δ ppm: 2.31 (s, 3H), 2.32 (s, 3H), 6.39 (d, *J* = 15.9 Hz, 1H), 7.25 (d, *J* = 8.4 Hz, 1H) 7.39 (d, *J* = 1.94 Hz, 1H), 7.43 (dd, *J* = 8.8 Hz, 2.2 Hz, 1H), 7.72 (d, *J* = 15.9 Hz, 1H). ^13^C NMR (176 MHz, CDCl_3_) δ ppm: 20.57, 20.62, 118.44, 122.98, 123.99, 126.67, 132.85, 142.46, 143.82, 145.00, 167.94, 168.06, 171.63.

4-O-Acetylferulic acid was synthesized applying the above procedure starting from FA (2.913 g, 15 mmol), DMAP (18 mg, 0.15 mmol), and acetic anhydride (1.684 g, 16.5 mmol). Purification procedure gave 4-O-acetylferulic acid (3.366 g, yield 95%). ^1^H NMR (700 MHz, CDCl_3_) δ ppm: 2.33 (s, 3H), 3.88 (s, 3H), 6.40 (d, *J* = 15.9 Hz, 1H), 7.08 (d, *J* = 8.0 Hz, 1H), 7.13 (d, *J* = 1.9 Hz, 1H), 7.15 (dd, *J* = 8.0, 1.9 Hz, 1H), 7.74 (d, *J* = 15.9 Hz, 1H). ^13^C NMR (100 MHz, CDCl_3_) δ ppm: 20.63, 55.95, 111.45, 117.11, 121.58, 123.36, 132.97, 141.86, 146.32, 151.49, 168.71, 170.48.

#### 2.4.2. Esterification of 4-O-Acetylsinapic Acid, 3,4-O-Diacetylcaffeic Acid, and 4-O-Acetylferulic Acid with β-Sitosterol

4-O-Acetylsinapic acid (2.130 g, 8 mmol) and β-sitosterol (2.903 g, 7 mmol) were placed in a three neck round bottom flask, equipped with thermometer, magnetic stirrer, and reflux condenser, under nitrogen atmosphere. Dichloromethane (50 mL) was added to the flask and the obtained solution was cooled to 0 °C in an ice bath. Next, DMAP (0.244 g, 2 mmol) and DCC (1.671 g, 8.1 mmol) were added sequentially to the solution. The reaction mixture was stirred for 30 min at 0 °C and 1 h at room temperature. Then, hexane (150 mL) was added and precipitated 1,3-dicyclohexylurea was removed by filtration. Solvents from the filtrate were evaporated under vacuum using rotary evaporator (Laborota 4003, Heidolph Instruments, Schwabach, Germany). The crude product was purified by flash column chromatography in glass column (Macherey-Nagel, Dueren, Germany) on silica gel using as an eluent: hexane/dichloromethane/ethyl acetate (3:1:1) to give β-sitosteryl (4-O-acetyl)sinapate (3.806 g, 82%). ^1^H NMR (700 MHz, CDCl_3_) δ ppm: 0.69 (s, 3H), 0.82 (d, *J* = 6.9 Hz, 3H), 0.84 (d, *J* = 6.7 Hz, 3H), 0.85 (t, *J* = 7.4 Hz, 3H), 0.93 (d, *J* = 6.5 Hz, 3H), 1.05 (s, 3H), 1.08–1.38 (m, 14H), 1.43–1.70 (m, 9H), 1.82–2.04 (m, 5H), 2.34 (s, 3H), 2.41 (m, 1H), 3.85 (s, 6H), 4.72–4.79 (m, 1H), 5.40–5.43 (m, 1H), 6.37 (d, *J* = 15.9 Hz, 1H), 6.77 (s, 2H), 7.60 (d, *J* = 15.9 Hz, 1H). ^13^C NMR (100 MHz, CDCl_3_) δ ppm: 11.87, 11.98, 18.79, 19.05, 19.34, 19.81, 20.42, 21.05, 23.09, 24.30, 26.12, 27.91, 28.25, 29.18, 31.90, 31.93, 33.96, 36.16, 36.64, 37.03, 38.24, 39.74, 42.33, 45.86, 50.07, 56.06, 56.19 (2 × OCH_3_), 56.71, 74.22, 104.65 (2 × CH), 119.02, 122.79, 130.36, 132.85, 139.62, 144.10, 152.42 (2 × C-OMe), 166.12, 168.46.

β-Sitosteryl (3,4-O-diacetyl)caffeate was synthesized applying the above procedure using 3,4-O-diacetylocaffeic acid (2.035 g, 7.7 mmol), β-sitosterol (2.903 g, 7 mmol), DCC (1.620 g, 7.85 mmol), and DMAP (0.214 g, 1.75 mmol). Purification on silica gel gave β-sitosteryl (3,4-O-diacetyl)caffeate (3.516 g, yield 76%). ^1^H NMR (400 MHz, CDCl_3_) δ ppm: 0.69 (s, 3H), 0.82 (d, *J* = 6.9 Hz, 3H), 0.84 (d, *J* = 6.9 Hz, 3H), 0.86 (t, *J* = 7.6 Hz, 3H), 0.93 (d, *J* = 6.6 Hz, 3H), 1.05 (s, 3H), 1.09–1.39 (m, 14H), 1.43–1.72 (m, 9H), 1.80–2.06 (m, 5H), 2.30 (s, 3H), 2.40 (m, 1H), 4.68–4.80 (m, 1H), 5.40 (d, *J* = 5.1 Hz, 1H), 6.36 (d, *J* = 15.9 Hz, 1H), 7.21 (d, *J* = 8.3 Hz, 1H), 7.35 (d, *J* = 2.2 Hz, 1H), 7.40 (dd, *J* = 8.4, 1.8 Hz, 1H), 7.60 (d, *J* = 15.9 Hz, 1H). ^13^C NMR (100 MHz, CDCl_3_) δ ppm: 11.86, 11.98, 18.78, 19.04, 19.33, 19.80, 20.59, 20.63, 21.04, 23.08, 24.29, 26.12, 27.86, 28.24, 29.18, 31.88, 31.92, 33.95, 36.15, 36.63, 37.02, 38.19, 39.74, 42.32, 45.85, 50.06, 56.05, 56.71, 74.28, 119.95, 122.69, 122.75, 123.86, 126.27, 133.44, 139.63, 142.41 (2 × C), 143.37, 165.95, 167.94, 168.00.

β-Sitosteryl (4-O-acetyl)ferulate was synthesized applying the above procedure using 4-O-acetylferulic acid (2.598 g, 11 mmol), β-sitosterol (4.147 g, 10 mmol), DCC (2.311 g, 11.2 mmol), and DMAP (0.305 g, 2.5 mmol). Purification on silica gel gave β-sitosteryl (4-O-acetyl)ferulate (5.443 g, yield 86%). ^1^H NMR (700 MHz, CDCl_3_) δ ppm: 0.69 (s, 3H), 0.81 (d, *J* = 6.7 Hz, 3H), 0.84 (d, *J* = 6.7 Hz, 3H), 0.85 (t, *J* = 7.4 Hz, 3H), 0.93 (d, *J* = 6.7 Hz, 3H), 1.05 (s, 3H), 1.08–1.38 (m, 14H), 1.42–1.71 (m, 9H), 1.82–2.04 (m, 5H), 2.32 (s, 3H), 2.41 (m, 1H), 3.86 (s, 3H), 4.72–4.78 (m, 1H), 5.41 (d, *J* = 5.2 Hz, 1H), 6.37 (d, *J* = 15.9 Hz, 1H), 7.04 (d, *J* = 8.2 Hz, 1H), 7.09–7.12 (m, 2H), 7.62 (d, *J* = 15.9 Hz, 1H). ^13^C NMR (100 MHz, CDCl_3_) δ ppm: 11.85, 11.98, 18.78, 19.04, 19.33, 19.80, 20.62, 21.04, 23.07, 24.29, 26.11, 27.88, 28.23, 29.16, 31.87, 31.91, 33.94, 36.15, 36.62, 37.02, 38.22, 39.73, 42.32, 45.85, 50.05, 55.88, 56.04, 56.69, 74.18, 111.18, 118.93, 121.18, 122.74, 123.20, 133.49, 139.63, 141.34, 143.66, 151.36, 166.17, 168.72.

#### 2.4.3. Deprotection of Acetoxy Groups

In a one neck round bottom flask equipped with reflux condenser and magnetic stirrer, β-sitosteryl (4-O-acetyl)sinapate (3.712 g, 5.6 mmol) was dissolved in a mixture of chloroform:methanol (2:1, 100 mL) and K_2_CO_3_ (0.152 g, 1.1 mmol) was added to this solution. The mixture was refluxed for 8 h, cooled to room temperature, and saturated aqueous NH_4_Cl (10 mL) was added. The mixture was diluted with dichloromethane (120 mL), washed with water (2 × 50 mL), saturated solution of NaCl in water (50 mL) dried with MgSO_4_, and filtered. Solvents were evaporated on rotary evaporator under vacuum and the crude product was purified by flash column chromatography on silica gel using as an eluent: hexane/dichloromethane/ethyl acetate (6:3:1) to give β-sitosteryl sinapate (3.025 g, yield 87%). ^1^H NMR (700 MHz, CDCl_3_) δ ppm: 0.69 (s, 3H), 0.82 (d, *J* = 6.7 Hz, 3H), 0.84 (d, *J* = 6.9 Hz, 3H), 0.85 (t, *J* = 7.4 Hz, 3H), 0.93 (d, *J* = 6.5 Hz, 3H), 1.05 (s, 3H), 1.08–1.37 (m, 14H), 1.43–1.70 (m, 9H), 1.81–2.04 (m, 5H), 2.40 (m, 1H), 3.92 (s, 6H), 4.72–4.78 (m, 1H), 5.41 (d, *J* = 5.2 Hz, 1H), 5.74 (s, 1H), 6.29 (d, *J* = 15.9 Hz, 1H), 6.77 (s, 2H), 7.58 (d, *J* = 15.7 Hz, 1H). ^13^C NMR (100 MHz, CDCl_3_) δ ppm: 11.85, 11.97, 18.78, 19.05, 19.31, 19.79, 21.04, 23.10, 24.29, 26.17, 27.94, 28.22, 29.21, 31.90, 31.92, 33.97, 36.15, 36.64, 37.05, 38.29, 39.75, 42.33, 45.88, 50.08, 56.08, 56.32 (2 × OCH_3_), 56.71, 73.95, 105.08 (2 × CH), 116.51, 122.69, 126.06, 137.10, 139.69, 144.64, 147.23 (2 × C-OMe), 166.47.

β-Sitosteryl caffeate was synthesized applying the above procedure using β-sitosteryl (3,4-O-diacetyl)caffeate (1.322 g, 2 mmol) and K_2_CO_3_ (0.108 g, 0.78 mmol). Purification on silica gel gave β-sitosteryl caffeate (0.762 g, yield 66%). ^1^H NMR (700 MHz, CDCl_3_) δ ppm: 0.69 (s, 3H), 0.82 (d, *J* = 6.9 Hz, 3H), 0.84 (d, *J* = 7.5 Hz, 3H), 0.85 (t, *J* = 7.4 Hz, 3H), 0.93 (d, *J* = 6.7 Hz, 3H), 1.05 (s, 3H), 1.07–1.37 (m, 14H), 1.40–1.68 (m, 9H), 1.82–2.04 (m, 5H), 2.39 (m, 1H), 4.70–4.76 (m, 1H), 5.40 (d, *J* = 5.0 Hz, 1H), 6.25 (d, *J* = 15.7 Hz, 1H), 6.87 (d, *J* = 8.4 Hz, 1H), 7.01 (dd, *J* = 8.2, 2.2 Hz, 1H), 7.07 (d, *J* = 1.9 Hz, 1H), 7.55 (d, *J* = 15.9 Hz, 1H). ^13^C NMR (75 MHz, CDCl_3_) δ ppm: 11.86, 11.98, 18.78, 19.04, 19.35, 19.81, 21.05, 23.07, 24.29, 26.09, 27.90, 28.25, 29.16, 31.89, 31.93, 33.95, 36.16, 36.64, 37.03, 38.24, 39.73, 42.33, 45.85, 50.04, 56.05, 56.71, 74.21, 114.38, 115.50, 116.31, 122.36, 122.73, 127.75, 139.67, 143.73, 144.47, 146.12, 167.04.

β-Sitosteryl ferulate was synthesized applying the above procedure using β-sitosteryl (4-O-acetyl)ferulate (5.380 g, 8.5 mmol) and K_2_CO_3_ (0.235 g, 1.7 mmol). Purification on silica gel gave β-sitosteryl ferulate (4.621 g, yield 92%). ^1^H NMR (700 MHz, CDCl_3_) δ ppm: 0.69 (s, 3H), 0.82 (d, *J* = 6.7 Hz, 3H), 0.84 (d, *J* = 6.9 Hz, 3H), 0.85 (t, *J* = 7.4 Hz, 3H), 0.93 (d, *J* = 6.7 Hz, 3H), 1.05 (s, 3H), 1.07–1.38 (m, 14H), 1.43–1.70 (m, 9H), 1.81–2.04 (m, 5H), 2.39 (m, 1H), 3.92 (s, 3H), 4.71–4.77 (m, 1H), 5.40 (d, *J* = 5.0 Hz, 1H), 5.85 (m, 1H), 6.28 (d, *J* = 15.9 Hz, 1H), 6.91 (d, *J* = 8.2 Hz, 1H), 7.03 (d, *J* = 1.7 Hz, 1H), 7.07 (dd, *J* = 8.2, 1.9 Hz, 1H), 7.60 (d, *J* = 15.7 Hz, 1H). ^13^C NMR (100 MHz, CDCl_3_) δ ppm: 11.86, 11.98, 18.78, 19.04, 19.34, 19.80, 21.04, 23.08, 24.30, 26.12, 27.93, 28.24, 29.17, 31.89, 31.92, 33.95, 36.15, 36.64, 37.05, 38.28, 39.74, 42.32, 45.85, 50.06, 55.92, 56.05, 56.70, 73.91, 109.27, 114.69, 116.12, 122.67, 123.01, 127.13, 139.73, 144.47, 146.74, 147.85, 166.64.

### 2.5. Addition of the Synthesized Steryl Hydroxycinnamates to Fat Samples

The refined rapeseed oil, mayonnaise and margarine were fortified with the purified β-SSA, β-SCA and β-SFA at four different concentrations: 0.01%, 0.02%, 0.1% and 0.5%. The HSEs were weighed accurately (0.01–0.5 g) and added to 100.0 g of oil transferred into an Erlenmeyer flasks and placed in a ultrasonic cleaner bath (Sono Swiss, SW 6H, Labo Plus, Warszawa, Poland) with ultrasound input power of 180 kW for 30 min due to the low solubility.

However, margarine samples with the same amounts of HSEs were melted into 25 mL glass jars and sonicated for 15 min in an ultrasonic cleaner bath. Furthermore, the same amounts (0.01–0.5 g) of HSEs and mayonnaise (100.0 g) were transferred into 25 mL glass jars and homogenized at 13500 rpm (homogenizer, DT basic, Yellow Line, IKA^®^-Werke GmbH & Co. KG, Staufen, Germany) for 30 s.

### 2.6. Antioxidant Activity Determination

#### 2.6.1. Samples Preparation

Before the AA determination of phenolic acids and their steryl esters, the fresh solutions of SA, CA and FA (c = 196.4–288.4 µmol/L) and HSEs (c = 354.4–1361.0 µmol/L) were prepared in methanol.

Moreover, the extracts of rapeseed oil and emulsions without and with the synthesized HSEs were obtained in methanol.

Briefly, 2.00 g of oil and 2.50 g of emulsions (margarine and mayonnaise) were weighed into test tubes and extracted with 5 mL of methanol for 30 min at ambient temperature in the dark using an orbital shaker (SHKA25081 CE, Labo Plus, Warszawa, Poland). The mixtures were frozen (−20 °C, 30 min) and the methanolic extracts were separated from fat samples. The extraction procedure was repeated three times for the same fat sample with fresh 5 mL portions of methanol. The resultant extracts were collected and stored into glass bottles in a refrigerator until the AA measurements were carried out.

#### 2.6.2. DPPH and ABTS Methods

Two spectrophotometric DPPH and ABTS methods described in our previous work [25] with some modifications were applied for AA determination of phenolic acids, synthesized HSEs, oil and emulsions before and after addition of HSEs at different concentrations (0.01–0.5%).

To determine the DPPH radicals scavenged, 0.5 mL of methanolic solutions of SA, CA, FA, β-SSA, β-SCA and β-SFA at various concentrations or 0.05–0.50 mL of oil and emulsion extracts were added to 1.5 mL and 1.95–1.5 mL of methanol, respectively and 0.5 mL of methanolic solution of DPPH (c = 0.30 mmol/L). The resultant mixtures were shaken vigorously and after 15 min of incubation at ambient temperature in the dark, the absorbance of each reaction mixture was measured at 517 nm against a reagent blank (2 mL of methanol + 0.5 mL of DPPH methanolic solution) by a spectrophotometer (Hitachi, Tokyo, Japan).

The ABTS^•+^ chromogenic radical reagent solution was prepared by reacting ABTS (7 mmol/L) with 2.45 mmol/L potassium persulfate (1:0.5) and kept for 16 h in the dark at ambient temperature before use. Ethanol was added to the blue–green solution to an absorbance was 0.70 at 734 nm. Various concentrations of the methanolic solutions of phenolic acids and HSEs (0.20 mL) as well as 0.025–0.20 mL of fat extracts were added to 2.30 and 2.425–2.30 mL of ABTS^•+^ solution and stand at 30 °C for 5 min. The absorbance was read at 734 nm against a reagent blank (2.5 mL of ABTS^•+^ solution).

The DPPH^•^ and ABTS^•+^ scavenging capacity were expressed as the concentrations of SA, CA, FA, β-SSA, β-SCA and β-SFA necessary to give a 50% reduction in the original absorbance. The 50% DPPH inhibition (IC_50(DPPH)_ values) and 50% ABTS inhibition (IC_50(ABTS)_ values) were calculated by linear regression analysis of dose–response curves plotting between the DPPH^•^ scavenging (%) or ABTS^•+^ scavenging (%) and concentrations.

Calibration curves were prepared on the same day (n = 5) using working solutions of Trolox (TE) in methanol between 0.02–0.10 µmol/mL and 0.01–0.15 µmol/mL for DPPH and ABTS methods, respectively. The least-squares method was applied to calculate the line’s equations: %DPPH = (707.00 ± 0.96) × c_TE_ + (2.46 ± 0.01) and %ABTS = (320.27 ± 3.51) × c_TE_ + (6.44 ± 0.01), resulting in determination coefficients, 0.9990 and 0.9990, respectively.

The AA of rapeseed oil, margarine and mayonnaise without and with HSEs were expressed as μmol of TE equivalents per 100 g of sample (μmol TE /100 g).

### 2.7. Statistical Analysis

The AA results of phenolic acids, steryl hydroxycinnamates as well as fat samples: refined rapeseed oil, margarine and mayonnaise before and after enrichment with the synthesized HSEs were evaluated by fivefold determination of each sample within the same day using the modified DPPH and ABTS methods.

The obtained AA results were expressed as: mean (c) ± standard deviation (SD). All data were statistically evaluated by analysis of variance (ANOVA) test. A post hoc Duncan’s test was applied for the calculation of the significant differences among mean AA values of the studied antioxidants and fat samples at probability level p < 0.05.

Statistical analysis of data was performed using the Statistica 8.0 software (StatSoft, Tulsa, OK, USA).

## 3. Results and Discussion

### 3.1. Synthesis of Steryl Hydroxycinnamates

**Scheme 1:**
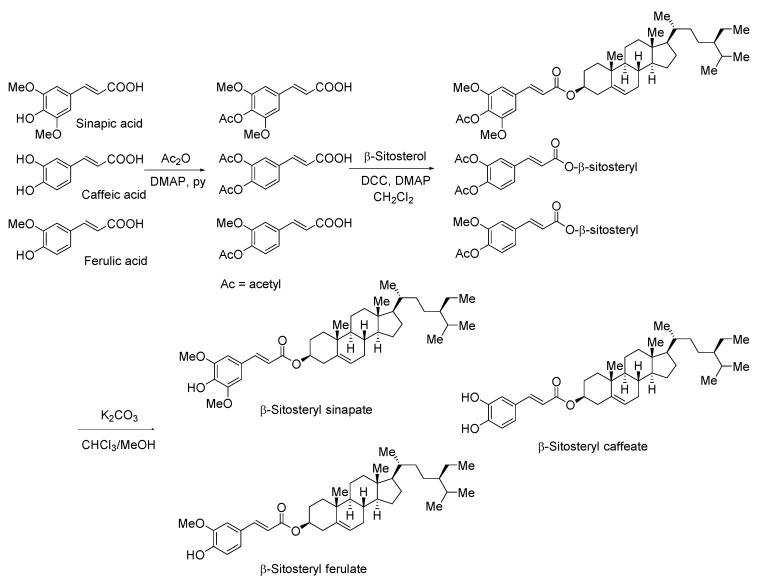
Synthesis of β-sitosteryl esters.

Syntheses of β-SSA, β-SCA and β-SFA were achieved through a chemical pathway (Scheme 1).

In previous work Winkler-Moser et al. [16] reported advantages of chemical approach for β-SFA synthesis due to the reduction of organic solvents (dichloromethane and toluene), shortening of synthesis time from overnight to a few hours, high yields and purity. Winkler-Moser et al. [16] improved chemical methods of β-SFA synthesis proposed by Ebenezer [26] and Condo et al. [27]. Constant interest in these FA derivatives caused a great interest in methods for the synthesis and practical use of these type of compounds e.g., reduction of synthesis time or toxic compounds. Currently, several approaches to β-SFA synthesis are known, especially enzymatic synthesis and chemical approach promoted by microwave irradiation with the characteristic steps: FA acetylation, esterification, and deprotection of acetoxy group [6,8]. The proposed synthesis technique was improved for time (esterification is a short process, 15 min.) and yield, while enzymatic synthesis was time consuming (over 120 h) [6]. It is known that the prolonging the reaction time caused the declination of esterification rate presumably due to decomposition of phytosteryl ester product. Additionally, better yields were observed for transesterification from methyl phenolic acid esters comparing to the direct esterification from phenolic acids. Therefore, high yield of enzymatic reaction requires additional steps or more expensive substrates [4]. On the other hand, enzymatic syntheses are desired in food industry to eliminate organic solvents and reagents.

Longer time of β-SCA synthesis, according to Winkler-Moser et al. [16] procedure, can be explained by the fact that there are two hydroxyl groups in aromatic ring of CA. However, low yield of the synthesized β-SCA was possible due to the loss of the product during the column chromatographic separation and the formation of by-products.

On the other hand, the commercially available β-sitosterol can be contaminated with other sterols, mainly campesterol and β-sitostanol. Therefore, there was minor signal characteristic for impurities on NMR spectra, although TLC plates did not demonstrate any fraction of impurities.

The steric hindrance of three groups in aromatic ring of SA (one hydroxyl and two methoxy groups) caused a decrease in yield of β-SSA synthesis in comparison with the synthesis yield of monomethoxylated β-SFA.

The modified chemical syntheses of β-SSA, β-SCA and β-SFA did not require a long time, were easily accessible and highly efficient, therefore can potentially be explored for the synthesis of other HSEs with biological activities.

### 3.2. Antioxidant Activity of the Synthesized Steryl Hydroxycinnamates

The AA of synthesized HSEs and phenolic acids analyzed by the DPPH and ABTS assays and the calculated IC_50_ results are presented in Table 1.

It is well known that ABTS and DPPH are technically simple, low-cost, spectrophotometric mixed mode assays based on single electron transfer (SET), hydrogen atom transfer (HAT) and proton coupled electron transfer (PCET) mechanisms frequently applied for determination of a sample’s free radical scavenging capacity. The ABTS method is suitable to determine the AA of both hydrophilic and lipophilic antioxidants in the same sample, while DPPH radical can only be dissolved in organic media, thus this assay has higher affinity toward lipophilic than hydrophilic antioxidants [28]. For this reason, these two analytical methods were chosen to compare the antioxidant potential of phenolic acids and their steryl esters having hydrophilic and hydrophobic character, respectively.

As can be seen, the mean results of IC_50(DPPH)_ and IC_50(ABTS)_ for three phenolic acids and their steryl esters differ significantly (Duncan test). The differences in chemical structures, mainly variation in number and arrangement of hydroxyl groups in aromatic ring as well as the different mechanisms of the used analytical methods and their different affinities toward hydrophilic and hydrophobic antioxidants affected the antioxidant potential of the analyzed compounds.

Among the studied compounds, phenolic acids were the most powerful antioxidants due to the lowest IC_50_ values (55.8–161.6 μmol/L), but their hydrophilic nature limited solubility in oils. On the contrary, β-SFA revealed the lowest antioxidant properties (IC_50(DPPH)_ = 290.0 μmol/L and IC_50(ABTS)_ = 206.0 μmol/L). This suggests that the hydrophilic CA was approximately 4 times more effective antioxidant than hydrophobic β-SFA. Although, the AA of β-SCA determined by DPPH assay (IC_50(DPPH)_ = 78.3 μmol/L) was higher than antioxidant potential of FA (IC_50(DPPH)_ = 161.6 μmol/L). However, the Duncan test indicated that ABTS values of FA and β-SCA were similar (Table 1). The structural modification of the carboxyl group in SA, CA and FA by esterification with β-sitosterol decreased the DPPH and ABTS of HSEs (approximately 40–125% and 50–110%, respectively) (Table 1). For this reason, significantly higher concentrations of β-SSA, β-SCA and β-SFA were required to scavenge 50% of the DPPH^•^ (IC_50_ = 78.3–290.0 μmol/L) and ABTS^•+^ (IC_50_ = 106.7–206.0 μmol/L) than the corresponding HCA (IC_50(DPPH)_ = 55.8–161.6 μmol/L and IC_50(ABTS)_ = 71.3–101.2 μmol/L].

On the other hand, CA and β-SCA as di-hydroxylated compounds are more efficient antioxidants than FA and β-SFA having only one hydroxyl group in the benzene ring due to greater resonance stabilization (Scheme 1). Furthermore, substitution of the hydroxyl groups at the 3- and 5-positions with methoxy groups in SA and β-SSA caused an increase of their DPPH and ABTS values (Table 1).

Satisfactory values of precision (relative standard deviation, RSD = 0.3–4.5%) demonstrate the benefit of the proposed DPPH and ABTS methods for the AA analysis of phenolic acids and their steryl esters.

For comparison, steryl sinapate and steryl ferulate ethylated enzymatically using *Rhizomucor miehei* lipase and transesterified by lipase from *Candida rugosa* had lower DPPH values in methanol (17.8–64.9%) than phenolic acids (DPPH_SA_ = 31.9–74.0% and DPPH_FA_ = 26.4–58.1%) used as suitable substrates for these reactions [4].

However, Winkler-Moser et al. [16] reported that much higher activity of FA measured by ABTS method (trolox equivalent antioxidant capacity (TEAC) = 1.155%) in comparison with the TEAC of steryl ferulates (0.762–1.094%) can be caused their worse solubility and steric hindrance prevented the steryl ferulates from reacting as efficiently with the ABTS^•+^.

On the contrary, conversion of SA, FA, CA and VA to their phytosteryl phenolate forms doubled their AA determined by ORAC method (ORAC = 8.83, 8.50, 5.81 and 4.00 μmol TE/μmol of phenolic acid samples, as well as 19.71, 13.99 and 8.54 μmol TE/μmol of phytosteryl phenolate samples). Moreover, ORAC value of phytosteryl sinapates (19.71 μmol TE/μmol of sample) was significantly higher than those for phytosteryl ferulates (16.49 μmol TE/μmol of sample), phytosteryl caffeates (13.99 μmol TE/μmol of sample) and phytosteryl vanillates (8.54 μmol TE/μmol of sample) [20,21,22]. This fact was explained by authors as the increase in hydrophobicity of phytosteryl phenolates compared with phenolic acids.

The obtained AA results of HSEs and their higher oil solubility demonstrated that synthesized antioxidants may have potential applications in food and pharmaceutical industries but future studies are needed to explain the reason for differences in the AA determined by different analytical methods.

### 3.3. Effect of Steryl Hydroxycinnamates Concentration on Antioxidant Activity of Real Fat Samples

Three commercial fat samples (refined rapeseed oil, margarine and mayonnaise) were spiked with different concentrations (0.01, 0.02, 0.1, and 0.5%) of the synthesized HSEs in order to evaluate their antioxidant efficacy by the modified DPPH and ABTS methods.

It is noteworthy that refined rapeseed oil had significantly higher antioxidant potential (DPPH = 390 μmol TE/100 g and ABTS = 1258 μmol TE/100 g) than the studied emulsions (DPPH = 212–267 μmol TE/100 g and ABTS = 811–856 μmol TE/100 g), whereas an insignificant difference for DPPH and ABTS results of margarine and mayonnaise samples were observed (Duncan test, Table 2).

Unfortunately, antioxidant potential of the investigated fat samples before and after addition of HSEs in the lowest concentrations of 0.01% and 0.02% (except DPPH of rapeseed oil with β-SCA) did not differ significantly (Table 2). These two levels of all synthesized HSEs had no antioxidant protective effect on w/o and o/w emulsions, whereas DPPH values increases with increasing β-SCA concentrations in the refined rapeseed oil. Furthermore, the DPPH and ABTS results of rapeseed oils enriched with 0.1% and 0.5% of HSEs were about 1.1–2.1-fold and 1.5–6.5-fold higher, respectively, than AA of refined rapeseed oil without the obtained steryl esters. In contrast, only addition of HSEs at the highest concentration of 0.5% caused the significant increase in radicals scavenging activity of the studied emulsions (Duncan test, Table 2). This suggests that all synthesized HSEs demonstrated good solubility in oil and emulsions at the highest concentration.

Nevertheless, differences in the effectiveness of antioxidants in emulsions are related to their properties such as polarity and partitioning as well as physical structure and rheological properties of the emulsions. The antioxidant efficacy can be changed by the localization of active compounds at the oil–water interface [18,29,30]. Therefore, the effectiveness of the studied steryl esters depended on the oil droplet characteristics (charge, size and composition) of mayonnaise and margarine. Although, pretreatment operations (heating, homogenization and sonication) during enrichment of emulsions with HSEs probably had profound effects on their physical structures.

The analyzed emulsions differed regarding their droplet size and surface composition that can affect the location of the added steryl esters, their surface activity and antioxidative capacity. Moreover, the concentrations of the added HSEs and the presence of natural antioxidants in the studied fat samples influenced their antioxidative activity and ability to protect lipids from oxidation. However, this hypothesis demonstrates the need for further studies in this area.

Interestingly, FA and steryl ferulates added to soybean oil at the lowest concentration (0.44 μmol/g), had not significant effect on its oxidative stability index (OSI = 7.89 h and 7.56–7.86 h for soybean oil without and with antioxidants, respectively), whereas the cholestanyl, cholesteryl, sitostanyl, sitosteryl and lanosteryl ferulates introduced to oil at higher concentrations (4.43 and 8.85 μmol/g) revealed a significant prooxidant activity (OSI = 8.60–8.61 h and 5.62–8.26 h for soybean oil without and with steryl ferulates, respectively) [16]. The prooxidant effect of steryl ferulates added to soybean oil at higher concentrations was explained by the fact that (1) oxidation products of steryl ferulates were generated and interacted with soybean triacylglycerols, (2) ferulic acid and oxidized ferulic acid released from steryl ferulates by hydrolysis can be acting as prooxidants, (3) the optimal concentration of the added esters was exceeded and they participated in reactions promoting oil oxidation.

The obtained DPPH and ABTS results for oil and emulsions fortified with steryl esters suggest that the synthesized compounds did not lose antioxidant efficacy at high concentrations.

The insignificant differences in radical scavenging activity of the supplemented margarine and mayonnaise samples determined by DPPH and ABTS assays can be explained by the similar mobility of the added steryl esters in the studied w/o and o/w emulsions, which affected their antioxidative effectivity (Duncan test, Table 2). Although, the AA of margarine and mayonnaise samples supplemented with β-SCA at a level of 0.1% analyzed by DPPH and ABTS assays increased by about 50–120% and 20% respectively. This fact indicated that β-SCA was more effective antioxidant at lower concentration (c = 0.1%) in comparison with β-SSA and β-SFA added to fat samples. The presence of two hydroxyl groups available to donate an electron or hydrogen atom confers the excellent antioxidant potential of β-SCA. The ability to transfer hydrogen of hydroxyl groups from the aromatic ring to DPPH and ABTS radicals confirms a decrease in antioxidant potential of the obtained HSEs in the following order: β-SCA > β-SSA > β-SFA.

For comparison, corn oil enriched with CA and its derivatives (phytosteryl caffeates and vinyl caffeates) had higher protection factors (PF = 1.5–2.0) than oil with SA, FA, VA and their esters (PF = 1.0) [23]. However, PF was less than 1.0 for corn oil after addition of FA and VA as well as their phytosteryl esters, hence these compounds revealed a slight pro-oxidative effect. Moreover, these authors reported that phytosteryl phenolates (except phytosteryl ferulates) were more effective antioxidants in o/w emulsions than phenolic acids and vinyl esters. Phytosteryl caffeates and sinapates had the highest AA with 55–57% inhibition of bleaching of β-carotene, while significantly lower inhibition for phytosteryl ferulates (20%) and phytosteryl vanillates (2.46%) was observed [23].

On the other hand, steryl sinapate and steryl ferulate retarded oxidation of bulk methyl linoleate less effectively than the free SA and FA, but addition of these two antioxidants (c = 1 μmol/g) decreased the formation of hydroperoxides during AA assay in emulsified methyl linoleate at 40 °C [4]. This indicated that antioxidative efficiency of the added steryl ester strongly depends on the type of food systems (low surface-to-volume ratio systems—bulk oil and high surface-to-volume ratio systems—emulsions), their hydrophilic or lipophilic character, other components, as well as its chemical structure, especially number and distribution of hydroxyl and/or methoxy groups in aromatic ring [18].

The calculated values of RSD (0.4–4.9%) indicate a reasonable repeatability of the DPPH and ABTS assays used to analyze the AA of rapeseed oil, margarine and mayonnaise samples before and after addition of the synthesized HSEs at concentrations between 0.01% and 0.5%.

## 4. Conclusions

Three steryl esters: β-SSA, β-SCA and β-SFA were successfully synthesized by a three steps chemical synthesis. High yields, 87%, 66% and 92% were achieved for esterification of β-sitosterol with SA, CA and FA, respectively. The specific chemical structures of the prepared HSEs were confirmed using spectroscopic analyses. The obtained steryl phenolates presented lower antioxidant potential determined by DPPH and ABTS than phenolic acids, but their higher oil solubility enabled their introduction to fat systems. Antioxidant properties of HSEs strongly depend on the chemical structures, especially presence, location and the number of hydroxyl and methoxy groups in aromatic ring.

The fortification of rapeseed oil, margarine and mayonnaise with lower concentrations (0.01% and 0.02%) of HSEs did not improve their radical scavenging activity determined by DPPH and ABTS methods. However, higher amounts (0.1% and 0.5%) of steryl phenolates in the investigated fat samples created effective defense system against free radical attack. Therefore, steryl phenolates, especially steryl caffeates, with the highest AA can be served as potential antioxidants in different food systems.

## Figures and Tables

**Table 1:** Antioxidant activity of phenolic acids and synthesized HSEs.

Antioxidant	IC_50(DPPH)_ ± SD (µmol/L)	IC_50(ABTS)_ ± SD (µmol/L)
SA	106.3 ± 0.3 ^c^	82.8 ± 0.3 ^b^
CA	55.8 ± 2.5 ^a^	71.3 ± 0.8 ^a^
FA	161.6 ± 0.5 ^d^	101.2 ± 1.0 ^c^
β-SSA	238.9 ± 6.9 ^e^	174.6 ± 6.7 ^d^
β-SCA	78.3 ± 1.5 ^b^	106.7 ± 4.1 ^c^
β-SFA	290.0 ± 5.4 ^f^	206.0 ± 7.9 ^e^

Values are means (n = 5) ± standard deviations (SD). Means with different letters (a–f) in a column are significantly different (*p* < 0.05) by the Duncan’s test.

**Table 2:** Antioxidant activity of refined rapeseed oil and emulsions before and after enrichment with different concentrations of the synthesized HSEs.

Sample	Concentration (%)	DPPH ± SD (µmol TE/100 g)	ABTS ± SD (µmol TE/100 g)
Oil	-	390 ± 13 ^e,f^	1258 ± 28 ^d,e,f^
RO + β-SSA	0.01	440 ± 13 ^g,h^	1277 ± 21 ^e,f^
	0.02	417 ± 10 ^f,g^	1241 ± 13 ^d,e^
	0.1	491 ± 5 ^h^	1412 ± 38 ^g^
	0.5	740 ± 3 ^k^	2321 ± 25 ^l^
RO + β-SCA	0.01	475 ± 14 ^h^	1238 ± 30 ^d,e^
	0.02	534 ± 10 ^i^	1269 ± 43 ^d,e,f^
	0.1	812 ± 20 ^l^	1952 ± 21 ^k^
	0.5	2526 ±18 ^o^	3451 ± 130 ^m^
RO + β-SFA	0.01	365 ± 2 ^d,e^	1221 ± 54 ^d,e^
	0.02	361 ±11 ^d,e^	1299 ± 7 ^e,f^
	0.1	464 ± 18 ^h^	1313 ± 11 ^f^
	0.5	630 ± 4 ^j^	1869 ± 14 ^j^
Margarine	-	212 ± 8 ^a,b^	811 ± 28 ^a^
MR + β-SSA	0.01	248 ± 12 ^a,b,c^	870 ± 15 ^a,b^
	0.02	244 ± 8 ^a,b,c^	859 ± 13 ^a,b^
	0.1	336 ± 13 ^d^	873 ± 25 ^a,b^
	0.5	456 ± 10 ^g,h^	1578 ± 19 ^h^
MR + β-SCA	0.01	204 ± 4 ^a^	880 ± 25 ^a,b^
	0.02	251 ± 7 ^a,b,c^	924 ± 23 ^b^
	0.1	459 ± 19 ^g,h^	1232 ± 40 ^d,e^
	0.5	1364 ± 22 ^m^	3492 ± 54 ^m^
MR + β-SFA	0.01	246 ± 8 ^a,b,c^	842 ± 17 ^a,b^
	0.02	215 ± 3 ^a,b^	897 ± 21 ^b^
	0.1	216 ± 9 ^a,b^	822 ± 30 ^a,b^
	0.5	459 ± 8 ^g,h^	1203 ± 24 ^d^
Mayonnaise	-	267 ± 10 ^b,c^	856 ± 42 ^a,b^
M + β-SSA	0.01	258 ± 4 ^b,c^	891 ± 11 ^b^
	0.02	233 ± 7 ^a,b^	847 ± 35 ^a,b^
	0.1	224 ± 3 ^a,b^	864 ± 12 ^a,b^
	0.5	329 ± 10 ^d^	1790 ± 16 ^i^
M + β-SCA	0.01	221 ± 4 ^a,b^	876 ± 25 ^a,b^
	0.02	233 ± 7 ^a,b^	877 ± 23 ^a,b^
	0.1	331 ± 2 ^d^	1044 ± 40 ^c^
	0.5	1644 ± 22 ^n^	4339 ± 54 ^n^
M + β-SFA	0.01	226 ± 8 ^a,b^	848 ± 17 ^a,b^
	0.02	281 ± 3 ^c^	855 ± 21 ^a,b^
	0.1	286 ± 9 ^c^	950 ± 30 ^b^
	0.5	478 ± 8 ^h^	1562 ± 19 ^h^

Values are means (n = 5) ± standard deviations (SD). Means with different letters (a–o) in a column are significantly different (*p* < 0.05) by the Duncan’s test.

## References

[1] Calheiros R., Machado N.F.L., Fiuza S.M., Gaspar A., Garrido J., Milhazes N., Borges F., Marques M.P.M. (2008). Antioxidant phenolic esters with potential anticancer activity: A Raman spectroscopy study. J. Raman Spectrosc..

[2] Decker E.A. (1998). Strategies for manipulating the prooxidative/antioxidative balance of foods to maximize oxidative stability. Trends Food Sci. Technol..

[3] Merkl R., Hrádková I., Filip V., Šmidrkal J. (2010). Antimicrobial and antioxidant properties of phenolic acids alkyl esters. Czech. J. Food Sci..

[4] Schär A., Liphardt S., Nyström L. (2017). Enzymatic synthesis of steryl hydroxycinnamates and their antioxidant activity. Eur. J. Lipid Sci. Technol..

[5] Garrido J., Gaspar A., Garrido E.M., Miri R., Tavakkoli M., Poural S., Saso L., Borges F., Firuz O. (2012). Alkyl esters of hydroxycinnamic acids with improved antioxidant activity and lipophilicity protect PC12 cells against oxidative stress. Biochimie.

[6] Begum A., Borah P., Chowdhury P. (2016). Microwave (MW) promoted high yield expedient synthesis of steryl ferulates—A class of novel biologically active compounds: A comparative study of their antioxidant activity with that of naturally occurring γ-oryzanol. Steroids.

[7] Nyström L., Moreau R.A., Lampi A.-M., Hicks K.B., Piironen V. (2008). Enzymatic hydrolysis of steryl ferulates and steryl glycosides. Eur. Food. Res. Technol..

[8] Schär A., Nyström L. (2016). Enzymatic synthesis of steryl ferulates. Eur. J. Lipid Sci. Technol..

[9] Zhu D., Brambilla D., Leroux J.-C., Nystrom L. (2015). Permeation of steryl ferulates through an in vitro intestinal barrier model. Mol. Nutr. Food Res..

[10] Mandak E., Nystrom L. (2012). Steryl ferulates, bioactive compounds in cereal grains. Lipid Technol..

[11] Aladedunye F., Przybylski R., Rudzinska M., Klensporf-Pawlik D. (2013). γ-Oryzanols of North American wild rice (*Zizania palustris*). J. Am. Oil Chem. Soc..

[12] Fang N.B., Yu S.G., Badger T.M. (2003). Characterization of triterpene alcohol and sterol ferulates in rice bran using LC-MS/MS. J. Agric. Food Chem..

[13] Takagi T., Iida T. (1980). Antioxidant for fats and oils from canary seed—sterol and triterpene alcohol esters of caffeic acid. J. Am. Oil Chem. Soc..

[14] Wen-Sen H., Hanyue Z., Zhen-Yu C. (2018). Plant sterols: Chemical and enzymatic structural modifications and effects on their cholesterol-lowering activity. J. Agric. Food Chem..

[15] Nystrom L., Achrenius T., Lampi A.-M., Moreau R.A., Piironen V. (2007). A comparison of the antioxidant properties of steryl ferulates with tocopherol at high temperatures. Food Chem..

[16] Winkler-Moser J.K., Hwang H.-S., Bakota E.L., Palmquist D.A. (2015). Synthesis of steryl ferulates with various sterol structures and comparison of their antioxidant activity. Food Chem..

[17] Wang T., Hicks K.B., Moreau R. (2012). Antioxidant activity of phytosterols, oryzanol, and other phytosterol conjugates. J. Am. Oil Chem. Soc..

[18] Laguerre M., Bayrasy C., Panya A., Weiss J., McClements D.J., Lecomte J., Decker E.A., Villeneuve P. (2015). What makes good antioxidants in lipid-based systems? The next theories beyond the polar paradox. Crit. Rev. Food Sci. Nutr..

[19] Costa M., Losada-Barreiro S., Paiva-Martins F., Bravo-Díaz C., Romsted L.S. (2015). A direct correlation between the antioxidant efficiencies of caffeic acid and its alkyl esters and their concentrations in the interfacial region of olive oil emulsions. The pseudophase model interpretation of the ‘‘cut-off’’ effect. Food Chem..

[20] Tan Z., Shahidi F. (2013). Phytosteryl sinapates and vanillates: Chemoenzymatic synthesis and antioxidant capacity assessment. Food Chem..

[21] Tan Z., Shahidi F. (2012). A novel chemoenzymatic synthesis of phytosteryl caffeates and assessment of their antioxidant activity. Food Chem..

[22] Tan Z., Shahidi F. (2011). Chemoenzymatic synthesis of phytosteryl ferulates and evaluation of their antioxidant activity. J. Agric. Food Chem..

[23] Tan Z., Shahidi F. (2013). Antioxidant activity of phytosteryl phenolates in different model systems. Food Chem..

[24] Ming-Ming Z., Lian W., Feng-Hong H., Ling D., Ping-Mei G., Qian-Chun D., Wen-Lin L., Chang Z. (2012). Ultrasonic pretreatment for lipase-catalyed synthesis of phytosterol esters with different acyl donors. Ultrason. Sonochem..

[25] Szydłowska-Czerniak A., Rabiej D., Krzemiński M. (2018). Synthesis of novel octyl sinapate to enhance antioxidant capacity of rapeseed–linseed oil mixture. J. Sci. Food Agric..

[26] Ebenezer W.J. (1991). Synthesis of 2880-II, a metabolite related to ferulic acid. Synth. Commun..

[27] Condo A.M., Baker D.C., Moreau R.A., Hicks K.B. (2001). Improved method for the synthesis of trans-feruloyl-β-sitostanol. J. Agric. Food Chem..

[28] Apak R. (2019). Current issues in antioxidant measurement. J. Agric. Food Chem..

[29] Jacobsen C., Hartvigsen K., Lund P., Meyer A.S., Adler-Nissen J., Holstborg J., Hølmer G. (1999). Oxidation in fish-oil-enriched mayonnaise 1. Assessment of propyl gallate as an antioxidant by discriminant partial least squares regression analysis. Eur. Food Res. Technol..

[30] Jacobsen C., Hartvigsen K., Lund P., Adler-Nissen J., Hølmer G., Meyer A.S. (2000). Oxidation in fish-oil-enriched mayonnaise 2. Assessment of the efficacy of different tocopherol antioxidant systems by discriminant partial least squares regression analysis. Eur. Food Res. Technol..

